# Association between genetic mutations in lung adenocarcinoma and adult body mass index: a retrospective cohort study

**DOI:** 10.3389/fonc.2025.1661143

**Published:** 2025-09-22

**Authors:** Yan qiao Wu, Lei Yang, Depeng Jiang

**Affiliations:** Department of Respiratory Medicine, The Second Affiliated Hospital of Chongqing Medical University, Chongqing, China

**Keywords:** lung adenocarcinoma (AD), body mass index, gene mutation, restricted cubic spline curve, logistic regression

## Abstract

**Background:**

The impact of obesity on gene mutations in lung adenocarcinoma(ADC) remains unclear. This study investigates the association between Body Mass Index(BMI) and the gene mutation in ADC.

**Methods:**

This study investigated the relationship between BMI and ADC gene mutation. Logistic regression model was used to verify the association between BMI and ADC gene mutations. Threshold effect analysis is used to distinguish between linear and nonlinear. Subgroup analyses rigorously assessed demographic variations, significantly strengthening the study’s credibility. Concurrently, the ROC curves were utilized to predict ADC gene mutation.

**Results:**

In our study, average ages of 66.00 *vs*. 65.00 were observed for ADC with gene mutation and ADC without gene mutation, respectively. After adjusting for covariates, a significant negative association emerged between BMI and ADC gene mutation, revealing a linear association(OR = 0.820, 95% CI: 0.680-0.982, p = 0.030). ROC curve analysis demonstrated AUC values of 0.533(all BMI) and 0.625(Q2, 21.08 ≤ BMI < 22.86kg/m^2^) for predicting tumor gene mutation. Spearman’s analysis further indicated a negative correlation between tumor gene mutations and BMI(Q2, OR= -0.215, p = 0.032). Patients younger than 60 years of age, women and those without a history of smoking and drinking had a relatively high rate of ADC gene mutations.

**Conclusion:**

This study reveals a significant association between BMI and ADC gene mutations, demonstrating that elevated BMI levels correlate negatively with mutation risk within the specific range of 21.08 to 22.86 kg/m². Furthermore, the rate of gene mutation may be relatively high in women under the age of 60 who do not smoke or drink. This study may assist clinicians in preliminarily assessing the gene mutation status of patients with ADC.

## 1 Introduction

Overweight and obesity constitute a major public health emergency, presenting a grave and imminent threat to global health progress. In 2021 alone, these conditions contributed to 71 million deaths and 129 million disability-adjusted life years (DALYs). Over the past two decades, the global age-standardized DALY rates linked to overweight and obesity have risen by more than 15%, making them one of the foremost risk factors and showcasing the steepest increase in disease burden attributable to any cause (1, 2).

Lung cancer is the most common malignancy worldwide, with an estimated 2.48 million new cases and 1.82 million deaths reported globally in 2022. By 2050, projections suggest that China alone will see approximately 1.12 million new cases and 960,000 deaths among males, alongside 680,000 new cases and 450,000 deaths among females (3, 4). Non-small cell lung cancer (NSCLC) accounts for roughly 85% of all lung cancers, with EGFR mutations occurring in 40% to 60% of Asian patients diagnosed with ADC (5).

Obesity is closely linked to tumor development, with cancer cases attributed to obesity projected to surpass 2 million globally by 2070, accounting for 7% of all cancer cases (6). Another study have demonstrated that the obesity-related gene ABCC8 may promote the development of hepatocellular carcinoma (HCC) (7). However, previous studies analyzing molecular data from human and mouse samples revealed that obesity-induced microenvironmental changes, rather than novel driver gene mutations, drive the progression of pancreatic ductal ADC (8). A critical discovery was the remarkable adaptation of islet cells within tumors associated with obesity. Moreover, the tumor microenvironment plays a key role in the development and progression of tumors, and its interaction with genetic mutations directly affects the invasiveness and metastatic potential of the tumor (9–11). Microenvironmental factors are universal across various types of tumors (12–14). In ADC, microenvironmental components such as immune cell infiltration, angiogenesis, and extracellular matrix remodeling often synergize with specific mutations to exacerbate the malignant phenotype of the tumor (15, 16).

However, research on the link between obesity and the gene mutation rate in ADC remains limited. The main goal of this study is to comprehensively investigate the relationship between obesity and gene mutation rate in ADC patients. To achieve this objective, genetic testing data from 404 clinically confirmed ADC cases were analyzed. The results indicate that higher BMI levels are associated with a reduced likelihood of gene mutations. This research is expected to offer new insights and potential avenues for future research into tumor gene mutations.

## 2 Materials and methods

### 2.1 Research participants

**Figure 1:**
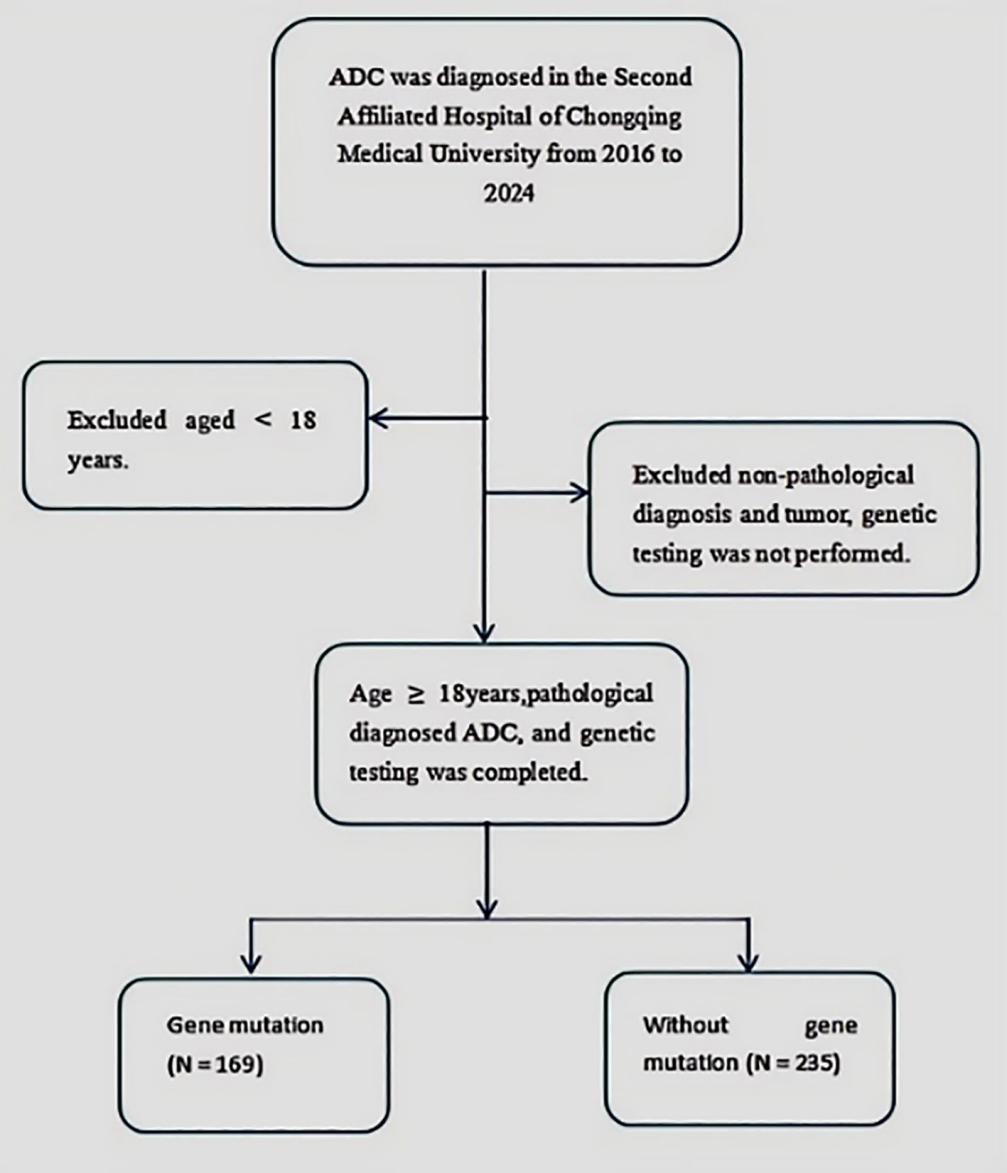
Flowchart illustrating the process of participant selection. ADC, Adenocarcinoma.

A retrospective, single-center study was conducted on patients diagnosed with ADC at the Second Affiliated Hospital of Chongqing Medical University from 2016 to 2024. The inclusion criteria required participants to be aged 18 years or older, have a pathologically confirmed ADC diagnosis, and have undergone complete tumor genomic profiling. Patients under 18 years of age or with incomplete genomic profiling results were excluded. The detailed selection process is presented in **Figure 1**. Relevant clinical data were systematically extracted from comprehensive electronic medical records.

### 2.2 ADC gene testing

For tumor mutation gene detection, all participants underwent Next Generation Sequencing (NGS), with all analyses conducted in laboratories within China. Whole-exome NGS DNA libraries were prepared using the NEBNext^®^ Ultra™ DNA Library Prep Kit in combination with the TruSeq Exome Enrichment kit, strictly following the manufacturer’s protocols. For targeted gene sequencing, a comprehensive panel covering 565 tumor-related genes was employed, utilizing TruSeq Custom Enrichment kits. Sequencing was performed on the high-throughput HiSeq X TEN platform. Raw sequencing data were processed with cutadapt to remove adapter sequences, and high-quality reads were accurately aligned to the human reference genome (hg19) using BWA (17). Somatic mutations were detected using MuTect, with high-confidence somatic SNVs identified based on stringent criteria (18). CNVs for each tumor sample were precisely assessed using ADTEx (19). Gene Ontology (GO) and Kyoto Encyclopedia of Genes and Genomes (KEGG) pathway enrichment analyses of the mutated genes were performed using KOBAS (20), with enriched terms defined as those with a False Discovery Rate (FDR) below 0.01. This study focused specifically on mutations in common genes, including Epidermal Growth Factor Receptor (EGFR), Kirsten Rat Sarcoma Viral Oncogene Homolog (KRAS), Tumor Protein p53(TP53), Anaplastic Lymphoma Kinase (ALK), Mesenchymal-Epithelial Transition factor (MET), and V-raf Murine Sarcoma Viral Oncogene Homolog B1 (BRAF).

### 2.3 Statistical methods

For ordinal variables, the median was used as the measure of central tendency. For non-normally distributed categorical variables, the Chi-square test or Fisher’s exact test was primarily applied, depending on the data structure. Qualitative parameters were analyzed using the Chi-square test, while quantitative data were assessed via ANOVA. To evaluate the association between BMI and ADC gene mutations, Multivariable Logit Models (I, II, III) were employed to calculate the odds ratio (OR) and 95% confidence interval (CI). Model I was unadjusted; Model II adjusted for age and sex; and Model III included additional adjustments for age, sex, smoking history, drinking history, hemoglobin, white blood cell count, platelet count, aspartate transaminase, alanine aminotransferase, creatinine, carcinoembryonic antigen, cytokeratin fragment 19, neuron-specific enolase, tumor location, and tumor type. The Spearman correlation test was used to assess the relationship between BMI and gene mutations. All statistical analyses were conducted using IBM SPSS (version 26; Armonk, NY), R 4.2.2, and Empowerstats 2.0 software, with GraphPad Prism (version 9.5.1) utilized for data visualization. Statistical significance was defined as a p-value < 0.05.

## 3 Results

### 3.1 General situation and basic characteristics

This study included 404 cases, comprising 235 with ADC gene mutations and 169 without. Notable differences between the mutated and non-mutated groups were observed for several variables: NSE levels (15.09 *vs*. 13.50, p = 0.024), gender distribution (69.82% *vs*. 48.09%, p < 0.001), smoking history (53.25% *vs*. 38.30%, p = 0.003), drinking history (36.69% *vs*. 26.38%, p = 0.027), nodule characteristics (73.37% *vs*. 84.26%, p = 0.009), and clinical stage (18.34% *vs*. 3.83%, p < 0.001). No significant differences were found for the other variables, as detailed in **Table 1**.

**Table 1:** General clinical characteristics of all participants.

Various	Without gene mutation(N = 169)	Gene mutation(N = 235)	P value
Age (years)	66.00(38.00-91.00)	65.00 (27.00-86.00)	0.137
Body Mass Index(kg/m^2^)	22.94(14.69-33.51)	22.66(13.78-30.30)	0.259
Hemoglobin(g/L)	126.00(70.00-163.00)	124.00(61.00-177.00)	0.327
White blood cell(10^9/L)	6.84(3.14-14.57)	6.71(2.89-15.81)	0.067
Platelet (10^9/L)	232.00(11.00-485.00)	216.00 (22.00-511.00)	0.100
Aspartate transaminase(10^9/L)	19.10(4.00-108.20)	18.00(4.00-115.00)	0.391
Alanine aminotransferase(10^9/L)	19.80(4.10-171.70)	20.00(4.00-269.00)	0.790
Creatinine(µmol/L)	63.30(5.00-127.80)	62.80(5.90-418.80)	0.619
Carcinoembryonic antigen	5.69(0.30-1500.00)	10.12(0.23-1000.00)	0.257
Cytokeratin Fragment 19	3.84(0.50-100.00)	3.29(0.71-100.00)	0.676
Neuron-Specific Enolase	15.09(6.98-83.54)	13.50(0.75-122.30)	0.024
Gender(%)			<0.001
Male	118(69.82%)	113(48.09%)	
Female	51(30.18%)	122(51.91%)	
Smoking history(%)			0.003
Yes	90(53.25%)	90 (38.30%)	
No	79(46.75%)	145(61.70%)	
Drinking history(%)			0.027
Yes	62(36.69%)	62(26.38%)	
No	107(63.31%)	173(73.62%)	
Tumor location(%)			0.815
Up left	41(24.26%)	51(21.70%)	
Low Left	31(18.34%)	46(19.57%)	
Upper right	47(27.81%)	62(26.38%)	
Center right	28(16.57%)	44(18.72%)	
Low right	10(5.92%)	20(8.51%)	
Hilus of the lung	12(7.10%)	12(5.11%)	
Tumor type(%)			0.009
Solid nodules	124(73.37%)	198(84.26%)	
Ground glass nodules	15(8.88%)	7(2.98%)	
Mixed nodules	30(17.75%)	30(12.77%)	
Tumor stage(%)			<0.001
I	31(18.34%)	9(3.83%)	
II	13(7.69%)	11(4.68%)	
III	25(14.79%)	14(5.96%)	
IV	100(59.17%)	201(85.53%)	

For continuous variables, utilize the Kruskal-Wallis rank sum test; for categorical variables (or counting variables), employ the Fisher’s exact test.

### 3.2 Association analysis between BMI and ADC gene mutation

After a thorough analysis of all pertinent variables, each unit increase in BMI corresponded to an 18% reduction in the ADC gene mutation rate (OR = 0.82, 95% CI: 0.680-0.982, p = 0.03). This robust evidence underscores a statistically significant inverse relationship between higher BMI levels and a decreased ADC gene mutation rate. Additionally, when examining BMI quartiles, individuals in Quartile 2 demonstrated a notable 63% lower odds of developing mutations compared to those in Quartile 1(OR = 0.365, 95% CI: 0.129-1.035, p = 0.05), as outlined in **Table 2**.

**Table 2:** Multivariable logistic regression models examining the correlation between BMI and ADC gene mutation.

Exposure	Model 1		Model 2		Model 3	
	OR (95%CI)	P-value	OR (95%CI)	P-value	OR (95%CI)	P-value
BMI index	0.826 (0.698,0.978)	0.026	0.824 (0.694,0.979)	0.028	0.817 (0.680,0.982)	0.031
BMI index quartile						
Q1	0.866 (0.667,1.125)	0.282	0.859 (0.651,1.135)	0.285	0.723 (0.497,1.053)	0.091
Q2	0.410 (0.182,0.923)	0.031	0.426 (0.187,0.973)	0.043	0.365 (0.129,1.035)	0.058
Q3	0.832 (0.422,1.640)	0.595	0.762 (0.374,1.551)	0.453	1.118 (0.421,2.971)	0.823
Q4	0.846 (0.669,1.070)	0.163	0.854 (0.672,1.086)	0.198	0.888 (0.646,1.219)	0.461

### 3.3 Analysis of RCS curve and threshold effects

**Figure 2:**
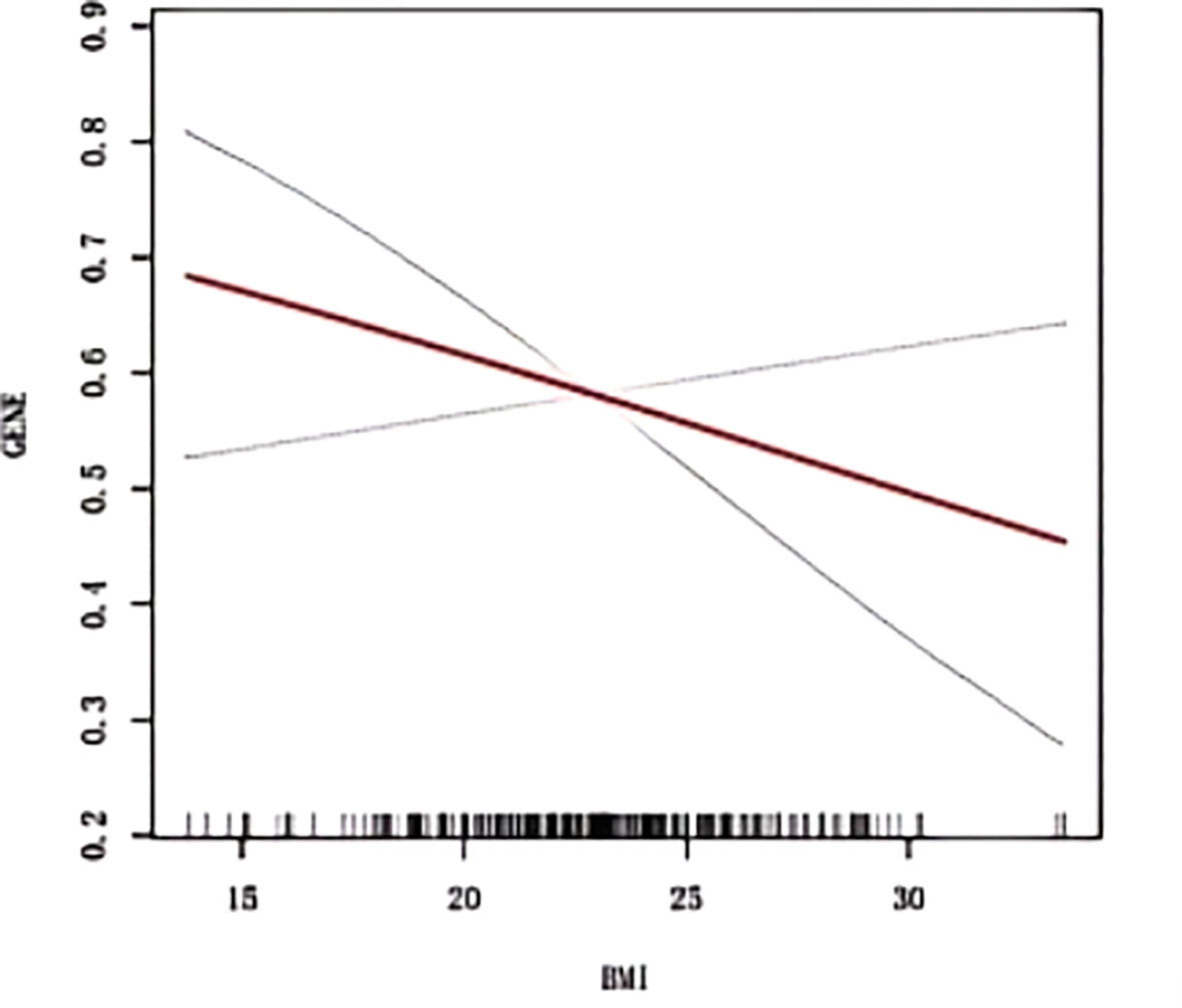
Smoothed curve fitting between BMI and ADC gene mutation. The blue bars show the fitted 95% confidence intervals(95% CI) and the fitted smoothed curves are shown in red. BMI, Body Mass Index; ADC, Adenocarcinoma.

The RCS curve in **Figure 2** vividly illustrates a notably strong linear relationship between BMI and ADC gene mutation rates. A detailed examination of the threshold effect reveals a pivotal turning point emerging at exactly 18.26 BMI units, as clearly shown in **Figure 2** and detailed in **Table 3**.

**Table 3:** The threshold effect analysis of BMI on ADC gene mutation.

	BMI the effect size (95%CI)	P value
Model 1 Fitting model by standard linear regression	0.953(0.886,1.025)	0.192
Model 2 Fitting model by two-piecewise linear regression		
Inflection point(K)	18.26	
< K	0.696(0.401,1.209)	0.198
> K	0.974(0.898,1.056)	0.520
P for likelihood ratio test	0.229	

BMI, Body Mass Index; ADC, Adenocarcinoma; OR, Odds Ratio, CI, Confidence Intervals.

### 3.4 Analysis of ADC gene mutations for age, sex, smoking, and drinking history

**Figure 3:**
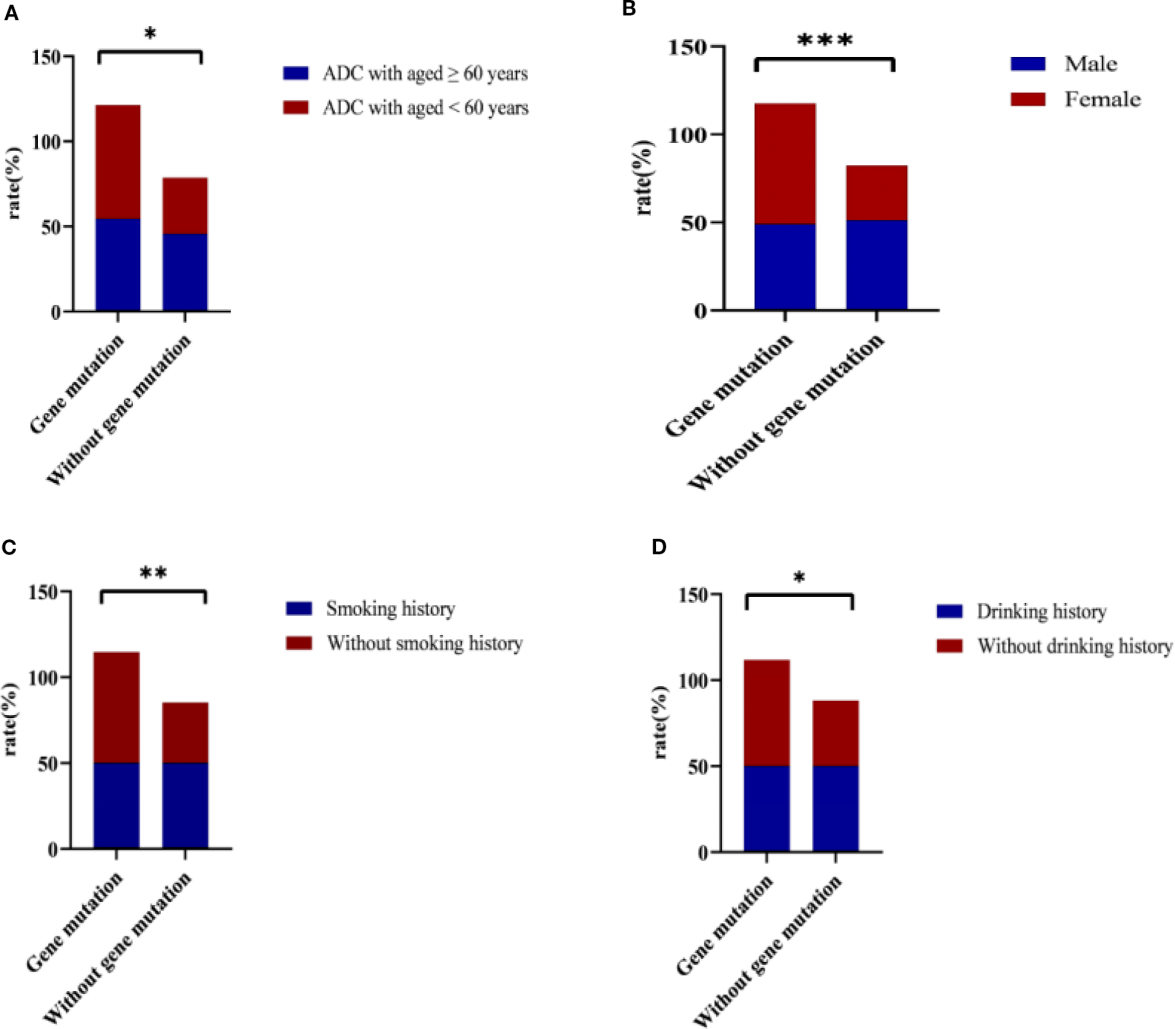
Gene mutations for age, sex, smoking and drinking. **(A)** Aged ≥ 60 or < 60 years; **(B)** Male and Female; **(C)** Smoking history; **(D)** Drinking history. ADC, Adenocarcinoma. * represent p < 0.05; ** represent p < 0.01; *** represent p < 0.001.

As depicted in **Figure 3A**, the ADC gene mutation rate was significantly lower in patients aged 60 years or older compared to those younger than 60 (54.4% *vs*. 66.9%, p = 0.019). Similarly, male patients exhibited a lower mutation rate than female patients (48.9% *vs*. 68.7%, p < 0.001), as depicted in **Figure 3B**. Furthermore, patients with a smoking history had a significantly lower mutation rate than non-smokers (50% *vs*. 64.7%, p = 0.003), as illustrated in **Figure 3C**. Likewise, the mutation rate was lower in patients with a drinking history compared to non-drinkers (50% *vs*. 61.8%, p = 0.027), as depicted in **Figure 3D**.

### 3.5 The ROC prediction for ADC gene mutation

**Figure 4:**
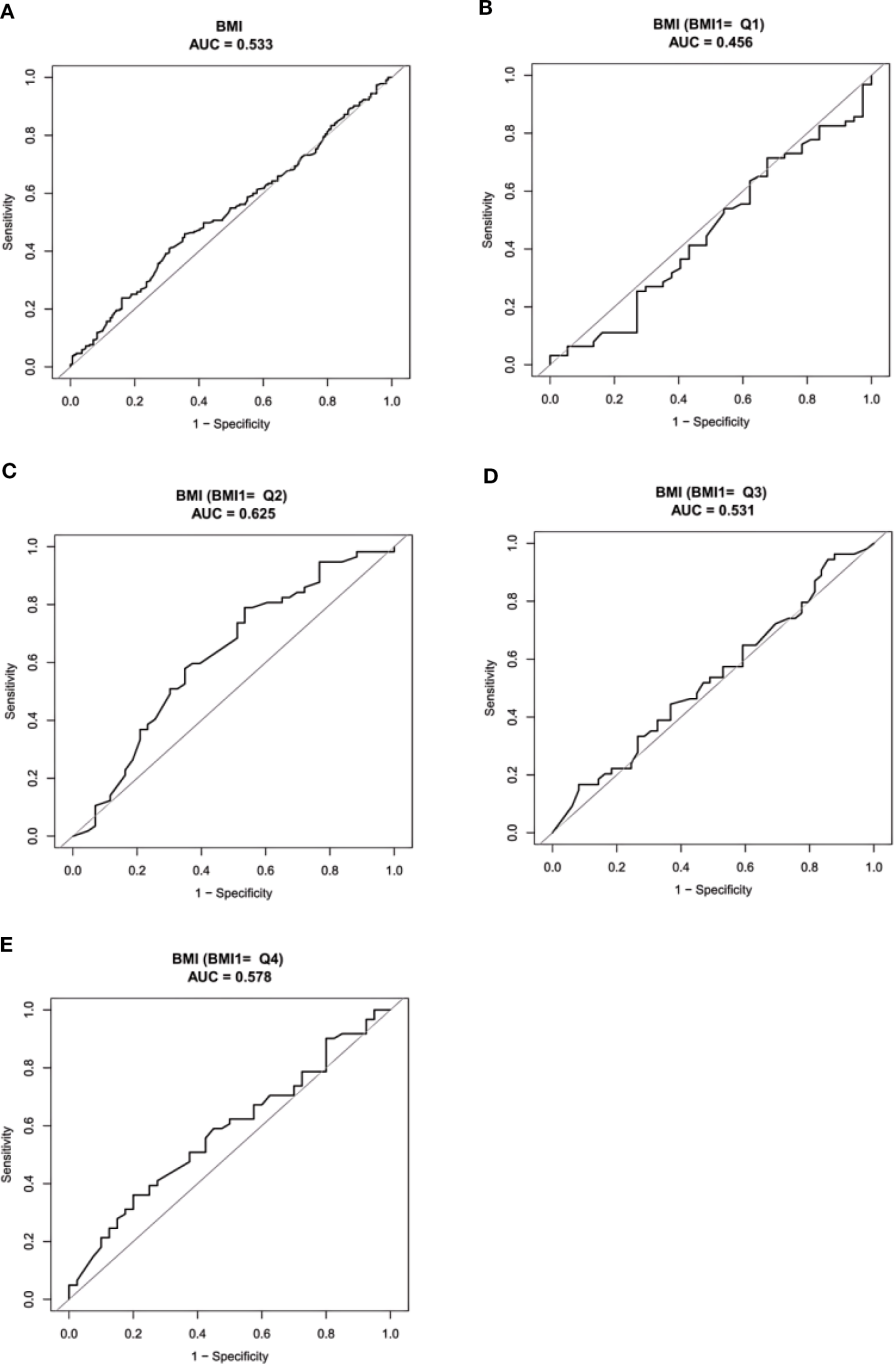
The ROC prediction of ADC gene mutation. **(A)** all BMI; **(B)** BMI < 21.08kg/m^2^; **(C)** 21.08 ≤ BMI < 22.86kg/m^2^; **(D)** 22.86 ≤ BMI < 24.74kg/m^2^; **(E)** BMI ≥ 24.74kg/m^2^. ADC, Adenocarcinoma; ROC, Receiver Operating Characteristic Curve; AUC, Area Under the Curve.

ROC analysis for predicting ADC gene mutations revealed the following AUC results: 0.533 for the overall BMI cohort (**Figure 4A**), 0.456 for individuals with BMI < 21.08kg/m^2^ (**Figure 4B**), 0.625 for those with 21.08 ≤ BMI < 22.86 kg/m^2^ (**Figure 4C**), 0.531 for 22.86 ≤ BMI < 24.74kg/m^2^ (**Figure 4D**), and 0.578 for BMI ≥ 24.74kg/m^2^ (**Figure 4E**).

### 3.6 The correlation analysis for BMI and ADC gene mutation

**Figure 5:**
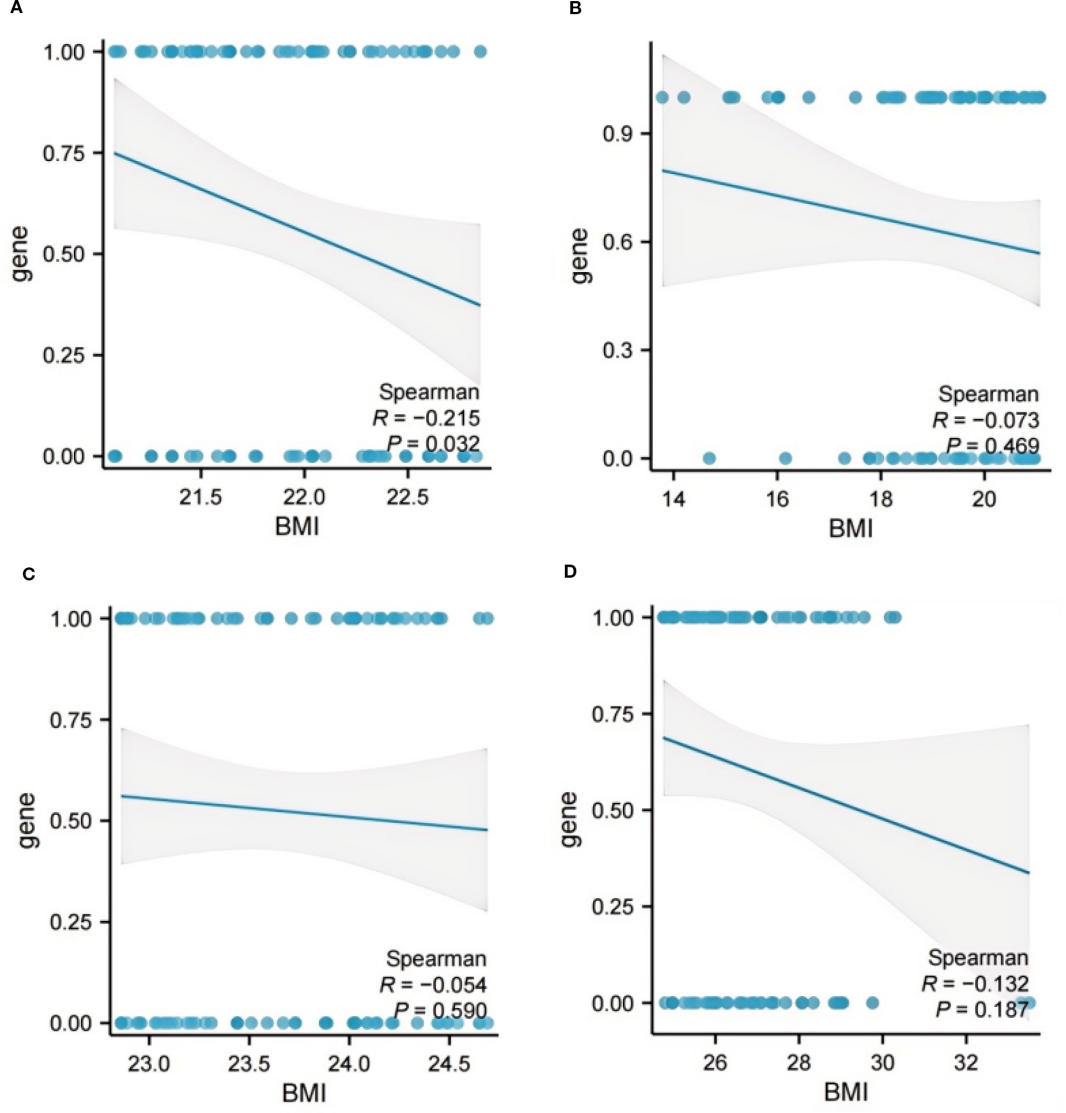
The correlation analysis between BMI and ADC gene mutation. **(A)** BMI < 21.08 kg/m^2^; **(B)** 21.08 ≤ BMI < 22.86 kg/m^2^; **(C)** 22.86 ≤ BMI < 24.74 kg/m^2^; **(D)** BMI ≥ 24.74kg/m^2^. ADC, Adenocarcinoma; BMI, Body Mass Index.

As depicted in **Figure 5A**, Spearman correlation analysis validated the relationship between BMI and ADC gene mutation. Specifically, within the BMI range of 21.08 ≤ BMI < 22.86kg/m^2^, a significant negative correlation was observed between BMI and ADC gene mutation (*R* = -0.215, p = 0.032). No significant correlations were identified in the other BMI quartile intervals (**Figure 5B–D**, all p > 0.05), with further details provided in **Supplementary Table 1**.

### 3.7 Stratified analysis

To comprehensively evaluate the consistency of the BMI-ADC gene mutation association and uncover potential variations across different subgroups, this study conducted extensive subgroup analyses and interaction tests, adjusting for multiple covariates, as detailed in **Table 4**.

**Table 4:** Subgroup analysis of the association between BMI and ADC gene mutation.

Various	OR(95%CI)	P value	P for interaction
Gender(%)			0.981
Yes	0.931(0.843,1.028)	0.158	
No	0.933(0.822,1.059)	0.281	
Smoking history(%)			0.739
Yes	0.926(0.826,1.039)	0.190	
No	0.932(0.838,1.037)	0.198	
Drinking history(%)			0.632
Yes	0.908(0.771,1.068)	0.243	
No	0.939(0.859,1.026)	0.165	
Tumor type(%)			0.359
Solid nodules	0.942(0.867,1.023)	0.153	
Ground glass nodules	0.019(0.000,Inf)	1.000	
Mixed nodules	1.094(0.859,1.392)	0.466	
Tumor location(%)			0.116
Up left	0.980(0.798,1.203)	0.845	
Low Left	0.844(0.659,1.080)	0.178	
Upper right	1.083(0.937,1.252)	0.279	
Center right	0.757(0.587,0.976)	0.032	
Low right	1.265(0.771,2.074)	0.352	
Hilus of the lung	0.510(0.000,Inf)	1.000	
Tumor stage(%)			0.690
I	28.622 (0.000, Inf)	0.999	
II	0.003(0.000, Inf)	1.000	
III	0.640(0.325,1.259)	0.196	
IV	0.982(0.891, 1.082)	0.713	

BMI, Body Mass Index, OR, Odds Ratio; CI, Confidence Intervals.

## 4 Discussion

Our study identified a significant linear association between BMI and ADC gene mutation rates, particularly notable within the BMI range of 21.08 to 22.86kg/m^2^. ROC prediction models for ADC gene mutations, based on BMI, yielded AUC values of 0.533 (all BMI) and 0.625 (BMI range: 21.08-22.86 kg/m^2^). Spearman correlation analysis further confirmed a significant negative correlation between BMI and ADC gene mutation rate in the range of 21.08 to 22.86 kg/m². Additionally, gene mutation rates were relatively higher among patients younger than 60, women, and those without a history of smoking or drinking.

Obesity is intricately linked to tumorigenesis, with underlying mechanisms that are notably complex. In obese individuals, dysfunctional adipocytes overproduce free fatty acids (FFAs) and pro-inflammatory cytokines, such as tumor necrosis factor-α (TNF-α) and Interleukin-6 (IL-6) (21). This excessive production activates the JNK/TLR/NF-κB signaling pathway, impairing insulin receptor function. Hyperinsulinemia subsequently promotes tumor cell proliferation through the PI3K-AKT-mTOR and RAS-MAPK pathways, while simultaneously reducing insulin-like growth factor-binding proteins (IGFBPs) and increasing the bioavailability of free Insulin-like growth factor-1/2 (IGF-1/IGF-2)—key factors for sustaining tumor stem cells (22–24). CD36, a crucial fatty acid transporter, is significantly upregulated in obesity and hypercholesterolemia, enabling gastric cancer cells to aggressively absorb lipids, thereby fueling rapid tumor growth and invasion (25). Notably, CD36 serves as the only shared genetic link between obesity, hypercholesterolemia, and gastric cancer, with its expression levels strongly correlating with advanced cancer staging and poor prognosis (26). Within obese adipose tissue, macrophages form a “crown-like structure”, continuously secreting pro-inflammatory factors that induce DNA damage and impair repair mechanisms. Furthermore, adipose tissue itself generates reactive oxygen species (ROS), further elevating mutation risks. Macrophages also polarize into the M2 phenotype, releasing inhibitory factors like Interleukin-10 (IL-10) and transforming growth factor-β (TGF-β), which suppress CD8^+^ T cell function and help cancer cells evade immune surveillance (27, 28).

Current understanding of modifiable risk factors for EGFR/KRAS-mutant ADC remains notably scarce. Prior research indicates that obese ADC patients have higher rates of KRAS mutations, and that KRAS-mutant tumors demonstrate increased responsiveness to immune checkpoint inhibitors, which partly explains the enhanced efficacy of immunotherapy in this group. Among East Asian populations, obesity exerts a more pronounced negative effect on EGFR mutations (29). Our study further uncovers a significant inverse relationship between BMI and ADC gene mutation rates, likely attributable to the high frequency of EGFR mutations within our cohort, underscoring the pivotal role of gene-environment interactions in ADC pathogenesis. Moreover, these findings highlight the potential clinical implications of incorporating BMI evaluations into genetic screening protocols for ADC patients, especially in populations with a high prevalence of EGFR mutations. Future investigations should explore the underlying biological mechanisms, such as hormonal or metabolic pathways, that facilitate the interaction between obesity and EGFR mutation prevalence. Such insights could pave the way for personalized prevention and treatment strategies. Additionally, longitudinal studies are needed to determine whether weight management interventions can mitigate mutation-associated risks and enhance immunotherapy outcomes in this specific patient subgroup.

Previous research has revealed significant differences in the mutation profiles of NSCLC, including alterations in genes such as ALK, Rearranged during Transfection (RET), MET, EGFR, serine/threonine kinase 11(STK11), Kelch-like ECH-associated protein 1 (KEAP1), BRAF, and KRAS, as well as in Tumor Mutation Burden (TMB)-High status, between patients under 40 and those aged 40 or older (30). Our study similarly demonstrates that ADC patients aged 60 or older have a significantly lower probability of gene mutations compared to those under 60. Furthermore, our findings indicate that women exhibit a lower rate of gene mutations than men, and patients with a smoking history have a markedly reduced mutation rate compared to non-smokers, which is consistent with the majority of prior studies (31–36). These observations underscore the substantial impact of age, gender, and smoking history on the likelihood of ADC gene mutations, providing a basis for optimizing screening and treatment strategies tailored to different age, gender, and smoking status groups. Further research should delve into the biological mechanisms associated with these factors, such as changes in DNA repair capacity, immune responses, or cell proliferation rates, which may influence the accumulation of gene mutations across diverse age, gender, and smoking contexts.

Various gene mutation types in ADC are strongly associated with a notably poor clinical prognosis. Patients with EGFR-sensitive mutations, such as exon 19 deletions or L858R, who are treated with first-generation EGFR tyrosine kinase inhibitors (TKIs), typically achieve a median progression-free survival (PFS) of 9 to 18 months (37). In contrast, first-line therapy with the third-generation TKI osimertinib extends median PFS to 18.9 months and overall survival (OS) beyond 3 years. Moreover, patients with exon 19 deletions have a more favorable prognosis than those with L858R mutations, with median PFS of 14.7 months versus 9.8 months, respectively. However, the presence of concurrent TP53 mutations significantly worsens prognosis, with a hazard ratio (HR) ranging from 1.6 to 2.3 (38). In the context of ALK fusion gene-targeted therapy, median OS exceeds 7 years, marking this subtype as having the most favorable prognosis (39). For MET exon 14 skipping mutations, targeted therapy yields an objective response rate (ORR) of 68% and a median PFS of 12.4 months (40). Nevertheless, the precise mechanisms contributing to the poor prognosis associated with distinct genetic mutation types remain incompletely understood. Future research should focus on multicenter prospective studies that incorporate single-cell sequencing and functional experiments to clarify the regulatory roles of signaling pathways, such as PI3K/AKT or MAPK, in various mutant subtypes, while these studies should evaluate the impact of the tumor immune microenvironment. A comprehensive analysis of multiple factors will provide a theoretical foundation for the development of personalized treatment strategies.

This study has notable strengths and limitations. Significantly, A key strength is that it is the first to explore the association between BMI and ADC gene mutations, including EGFR, KRAS, and TP53, potentially providing clinicians with fresh insights to inform evidence-based decision-making. However, several limitations warrant consideration. Firstly, as a single-center retrospective study with a relatively small sample size, it was not feasible to conduct a longitudinal analysis of the relationship between BMI and tumor gene mutations. Secondly, our research did not differentiate among specific gene mutations but rather examined the correlation between BMI and tumor gene mutation in a general sense. Thirdly, potential selection bias in the study data may affect the accuracy of the findings. Fourthly, the study population lacked racial diversity, which may limit the generalizability of the results. Finally, errors in data collection could introduce bias into the outcomes.

In summary, this retrospective real-world study reveals a significant inverse correlation between BMI and ADC gene mutations. Specifically, within the BMI range of 21.08 to 22.86 kg/m², higher BMI levels are associated with a reduced risk of such mutations. Furthermore, the mutation frequency appears to be elevated among female patients under 60 who have no history of smoking or alcohol consumption. These findings offer clinicians preliminary insights for assessing the likelihood of ADC mutations in patients.

## Data Availability

The original contributions presented in the study are included in the article/**Supplementary Material**. Further inquiries can be directed to the corresponding author.
