# Supplementary material for: Association between genetic mutations in lung adenocarcinoma and adult body mass index: a retrospective cohort study
Source: Front Oncol. 2025 Sep 22;15:1661143. doi: 10.3389/fonc.2025.1661143 (PMC12497579; doi:10.3389/fonc.2025.1661143)
Supplement: Supplementary file 1 [file Table1.docx]

| **Exposure** | **Outcomes** | **Method** | **Correlation** | **P value** |
| --- | --- | --- | --- | --- |
| BMI(all) | Gene mutation | Spearman | -0.056 | 0.260 |
| BMI(Q1) | Gene mutation | Spearman | -0.074 | 0.469 |
| BMI(Q2) | Gene mutation | Spearman | -0.215 | 0.032 |
| BMI(Q3) | Gene mutation | Spearman | -0.054 | 0.591 |
| BMI(Q4) | Gene mutation | Spearman | -0.132 | 0.187 |

Supplementary Figure 1. Spearman correlation analysis between BMI and ADC gene mutation. BMI, Body Mass Index.
